# Women’s experience and perspectives toward genital cosmetic surgery in Erbil city/Iraq: a qualitative study

**DOI:** 10.1186/s12905-022-01921-9

**Published:** 2022-08-12

**Authors:** Tiran Jamil Piro, Awaz Aziz Saeed, Warda Hassan Abdulla, Kolsoom Safari

**Affiliations:** School of Nursing and Midwifery, Hawler Medical University, Arbīl, Iraq

**Keywords:** Female genital cosmetic surgery, Female genitalia, Female sexuality, Sexual pleasure

## Abstract

**Background:**

Nowadays, Female Genital Cosmetic Surgery is quite prevalent, resulting in a wide range of medical and cultural implications. The majority of women who undergo this operation suffer from anxiety, depression, and other emotional symptoms. The present study was carried out in order to explore women’s perspective on and experience of genital cosmetic surgery given their special context and cultural aspects.

**Methods:**

The qualitative study applying content analysis examined the experiences of nine women who received genital cosmetic surgery in Erbil, Kurdistan-Iraq, between 2021 and 2022. Each of the semi-in-depth face-to-face interviews lasted between 40 and 60 min and was conducted at the physician's clinics.

**Results:**

Semi-structured interviews led to emergence of two main themes, namely “husband satisfaction” and “self-confidence”. Interpreting the participants’ stories resulted in some relevant subthemes and meaning units like “ugly appearance”, “anxiety related to husband undesirable intercourse”, and “dyspareunia”. Finally, the related conclusions of each theme were explored.

**Conclusion:**

As suggested by the study findings, female genital cosmetic surgery improves the women’s body image and sexual function and the couples’ sexual satisfaction, especially that of husbands. Because of the people’s sociocultural aspects in Kurdistan region, their awareness of female sexual needs and marital relationship needs to be raised.

## Introduction

Female Genital Cosmetic Surgery (FGCS), also known as vulvoplasty, refers to a surgical procedure through which the vulvovaginal anatomy is altered. This cosmetic surgery is generally performed for women with no apparent functional or structural abnormality, not for clinical purposes, such as urinary incontinence, vaginal prolapse, previous obstetric injury, or clinically diagnosed female sexual dysfunction [1].

Over recent years, FGCS has become quite popular, leading to an increase in a broad variety of medical and social implications. FGCS encompasses a group of non-medically indicated cosmetic surgical procedures which are aimed at altering the external and internal appearance and structure of female genitalia [2].

According to US 2013 statistics, labiaplasty with a rise of 44% in 2013 was reported to be the fourth most common cosmetic surgical procedure, following liposuction, breast augmentation and rhinoplasty [3]. From 2003 to 2013, the prevalence of labiaplasty increased 3 times in Australia and 4 times in the UK [4]. According to the statistics published by Australian government, requests for rebatable vulvoplasty increased from 640 in 2001 to more than 1500 in 2013, which is an increase of about 140% [5]. However, there has been no report on an associated increase in genital disease diagnoses [6]. An unknown number of surgeries are conducted in private hospitals and clinics; therefore, these figures cannot be the accurate number of vulvoplasties performed [5].

The Royal Australian College of General Practitioners published a resource guide for health professionals titled "Female Genital Cosmetic Surgery: A resource for general practitioners and other health professionals" on July 31, 2015, in response to the sharp increase in demand for FGCS and the need for guidelines regarding FGCS among general practitioners (GP). GPs are the first point of contact with the healthcare system; therefore, they have a significant and pivotal role in training girls and women regarding the various genital appearances and the risks associated with genital surgery. After vulvoplasty, most women experience anxiety, depression, and other psychiatric conditions; therefore, informed GPs can enhance the women’s health outcomes by tackling these modifiable psychosocial factors [5, 7]. In this regard, gynecologists and gynecological nurses need to be equipped with sufficient knowledge to recognize women with such complications [8, 9].

The present study was carried out in order to explore women’s perspective on and expectations of genital cosmetic surgery based on their context and cultural aspects. We discovered that there were little qualitative studies conducted on this topic in Muslim countries. In addition, we predicted a low participation rate due to the private nature of the topic, which comes from the women's cultural and religious perspectives. In light of this, it was determined that qualitative design would be the most suitable for this study.

## Patients and methods

### Research design

The present study adopted qualitative content analysis. Content analysis method is a subjective interpretation of the content of a text and is carried out by classifying the codes and identifying the themes. Content analysis is appropriate for the present research because it can easily accommodate a large amount of data. The collected data were analyzed through the approach introduced by Graneheim and Lundman (2004) [10]. Using this approach, the following steps were taken to analyze the collected data:

1. Transcribing the interviews verbatim and reading through the text several times to obtain a sense of the whole;
2. Dividing the text into condensed meaning units;
3. Abstracting the condensed meaning units and labelling with codes;
4. Sorting codes into categories and subcategories based on comparing their similarities and differences; and
5. Formulating themes as the expression of the latent content of the text.

### Study procedures

After permission was obtained from the interviewees, semi-structured face-to-face interviews were conducted with participants. Interviews were listened to several times and transcribed verbatim, and the data were studied in order to better understand the meaning units in the interviews and identify the extracted initial codes. According to the data analysis steps suggested by Graneheim and Lundman (2004), the researcher started dividing the texts into condensed meaning units and writing and reading texts carefully. In the next step, the condensed meaning units were abstracted, and labelling was done with codes. Later, the codes were sorted into categories and subcategories (e.g., ugly appearance, self-satisfaction, and love) based on comparing their similarities and differences. Ultimately, the main themes (i.e., husband satisfaction and self-confidence) were formulated as the expression of the latent content of the text. The accuracy and robustness of the data (i.e., validity, reliability, and transferability) were examined utilizing the criteria proposed by Cuba and Lincoln (1985) [11]. Investigating the validity and strength of qualitative studies is aimed at making sure that the participants’ experiences are exactly reflected, which necessitates adherence to accuracy in utilizing the citations and examining the phenomenon. Accuracy and clarity in explaining the phenomenon and conducting all phases of the study should ensure the reader that data record and other phases have been carried out accurately [12]. Four scales of credibility, dependability, conformability, and transferability related to qualitative studies are considered to be equivalent respectively to internal validity, reliability, objectivity, and external validity by qualitative researchers like Guba and Lincoln (1985) and Polite and Beck (2013) [11, 13]. Therefore, the present study employed the four criteria proposed by Guba and Lincoln to investigate the scientific accuracy of the results. For data validation, interaction with and closeness to the participants as well as peer review and comparison were taken into account. Data reliability was assessed by experts like head of higher education department and head of scientific committee because of their experience in nursing research.

### Sample size and sampling technique

Nine women who had experienced genital cosmetic surgeries in the city of Erbil, Kurdistan-Iraq were selected by the researchers through purposive sampling. This research tried to include individuals with maximum diversity regarding age and type of cosmetic surgery. The inclusion criteria for the study sample selection were women who had undergone genital cosmetic surgery at least three months before the initiation of the study, those with possibility of access to their addresses and phone numbers, those who were completely vigilant at the time of data collection, and those who were willing to participate in the study. Exclusion criteria were the women who refused to participate in the study. The participants were selected after consulting with 2 gynecologists who did most of the genital cosmetic surgeries in Erbil city. They gave their patients’ names and phone numbers to the researchers. Afterwards, the researchers phoned them and set time and place for the in-depth interviews. All interviews were conducted in the physicians’ clinics, and each took 40 to 60 min depending on the participants’ eager, speaking ability and tolerance. The interviews were recorded after their consent was obtained and included open ended questions like “What was your reason to do genital cosmetic surgery?”, “What has been the effect of cosmetic surgery on your life?”, “Can you tell me about your experience of genital cosmetic surgery”. Depending on their responses to the first questions, follow-up questions like “Could you please explain more?”, “Did you mean this….?”, and “If it is possible, give some examples” were asked. It should be noticed that the number and sequence of the questions differed in different interviews. The type of the questions was arranged based on the participants’ experiences, abilities and understanding of the issue, and the progress of the interviews. Also, more relevant questions were employed in order to better conduct the interviews and reach data richness. The sample size was based on data saturation and redundancy such that there was no need for further interview [14]. Sample selection was found to be a difficult task because most of the cases refused to participate in the study due to privacy and personal concerns. Another issue was that there were a few cases in the units or clinics of genital cosmetic surgery. Also, some of the participants were not willing to answer some of the researchers’ questions, and sometimes they provided short answers. Informed consent was obtained from all participants. The participants were assured that they will be anonymous, that they will be free to abandon the interview at any time, and that they can avoid answering any questions. This process has been done by two authors of this study. The reminder (including corresponding author) participated in the process of data analyzing, writing, and translating.

### Findings of the study

The main themes emerged from interviews with participants to understand about their reasons for and perspectives on genital cosmetic surgery, including "husband satisfaction" and "self-confidence." Moreover, relevant subthemes and meaning units were extracted from the direct quotations which were retrieved from the participants’ stories, and the related conclusions of each theme were explored. As mentioned previously, the participants aged between 21 to 50 years, 7 of them were employed and office employees, the remainder were housewives (Table1). Seven women were married, one was divorced but had a fiancé, and one of them was unmarried.

**Table 1:** The participants’ demographic characteristics

Participants	Age	Marital status	No. children
1	27	Married	5
2	50	Married	7
3	24	Engaged	0
4	23	Single	0
5	21	Married	0
6	50	Married	7
7	30	Divorce	3
8	23	Married	1
9	48	Married	5

### The first main theme: husband satisfaction

Based on the data extracted from the interviews, the main theme was “Husband Satisfaction” which included three subthemes, namely “ugly appearance”, “anxiety related to husband undesirable intercourse”, and “dyspareunia”.

The majority of the participants complained about their husband’s dissatisfaction with their intercourse, which made them feel anxious. They also stated that this condition made their relationship with their husband problematic, which in turn had negative effects on their lives. Moreover, they were anxious about their marital relationship and satisfying their husband’s specific needs. As they revealed, the ugly genital appearance and dyspareunia were two causes of this problem, which increased their anxiety related to husband undesirable intercourse. For example, Participant No. 1 who aged 27 years and had 5 children stated:

“After my first childbirth, my genital area tore, but the midwife didn’t suture it, so I frequently experienced infection for nearly 7 years. Especially, I felt that gases came out of my vagina during intercourse, and my husband always complained about its appearance and grumbled. My physician said that some women do such interventions just to change their body image. She suggested me surgery as the best way to get rid of my problem. Now after genital repair and such surgery, my partner is satisfied, but I’m in pain during intercourse”.

In the same regard, Participant No. 2 who was 50 years old mentioned:

“During my last delivery, I had laceration in my vagina and vulva. My midwife sutured it, but it opened and caused pain and infection after two times of repairing, especially during intercourse. Now I reject to repair it again because I have no luck. My husband complains, but I cannot change the condition, what can I do?”

Moreover, Participant No. 5 mentioned a wonderful subject which reflected the subtheme of ugly appearance as a result of female circumcision or delivery. In this regard, she narrated:

“I was married, but neither my husband nor I was satisfied with our intercourse, because when I was a small girl, my parents took me to a circumciser, and she cut half of my genital labia in an irregular shape. After the surgery, my husband’s complaints decreased without notifying myself like some Kurdish women. We should remain silent and say yes to our husbands all the time”.

Furthermore, Participant No. 6 who aged 50 years with 7 children related:

“Throughout all of my married life, I was unhappy with our intercourse because my husband was always nagging to me and said I was like a dry stick because I am one of hundreds of girls who was circumcised during childhood, which I thought was one of the reasons for his nagging. He closed his eyes to my real world as a woman and human and didn’t understand my condition. Anyway, I’m better now because I did surgery and I feel better”.

As revealed by the participants, women’s ability to satisfy their spouses effected their married lives and relationship. They tried to retrieve the normal shape and size of their genital organ to satisfy their spouse’s desire.

### The second main theme: self-confidence

Some of the participants revealed their experience of prolapse of uterus or collection of fatty tissue in the genital area after delivery, leading to the extraction of subthemes, entitled “self-satisfaction” and “love and attention”. In this regard, one of the participants who was a 50-year-old woman talked about having pain and discomfort which had led to delivery complications and made her attend the gynecologist for surgery and repair her perineal area to improve comfortability for normal life and intercourse so as to gain her partner’s attention and love to increase self-satisfaction. She further added:

“After my childbirth, my uterus came down, so that I could feel it when I sat. This condition made me uncomfortable too much. I had pain which led to both uterine prolapse and hemorrhoid. To resolve my complications, I did surgery, and now I am satisfied with sexual activity with my husband. Unlike the past, I think he loves me more because he speaks with me more cordially”.

Participant No. 7 who was divorced, aged 30 years, and had 3 children wanted to marry again, so she prepared herself for marriage by doing some plastic and cosmetic surgery to remove her fatty tissue from her abdomen and genital area following her fiancé’s request. Hymen is culturally very important for both partners, and it increases self-satisfaction and gaining self-confidence especially during their first intercourse. In this regard, she stated:

“After the proposal and engagement, my fiancé asked me to repair my genitalia, especially my hymen through hymenoplasty to be like before my first marriage. So, after I attended a physician, she repaired my vagina and did hymenoplasty. Now I feel more confident, and I’m ready for marriage”.

Participant No. 4 who was an unmarried 23-years-old girl stated:

“I want to marry, but I was thinking of doing labiaplasty before marriage, so I did it because I experienced circumcision during my childhood, leading to irregular size of my labia.”

Regarding love and attention after genital repairing, Participant No. 9 that aged 48 years revealed:

“I’ve got 5 children. After delivery of each of them, I had many problems with urination and intercourse. My husband complained about dissatisfaction with our intercourse. I felt that he didn’t love me, because of our sexual problems. Those problems affected our married life and decreased my self-steam, especially during intercourse.”

In the same regard the same participant stated:

“Immediately after my last childbirth I asked my physician to repair my perineal area, because it was the main reason for marriage problems between me and my husband after our previous childbirth. This cosmetic surgery raised love between us. I believe if the woman fails to satisfy her husband during sexual intercourse, they will face many problems in their married life”.

According to these narratives, Kurdish culture plays a significant role in marital life and dyadic relationships, as males are regarded as the dominating member of the family and women are expected to provide sexual satisfaction for their husbands while ignoring their own rights and desires.

## Discussion

As revealed by the results of analyzing the interviews, satisfying husbands and increasing self-confidence were found to be two significant reasons for the women’s efforts to change their body shape and size through genital cosmetic surgery. Also, it became known that their anxiety related to husband undesirable intercourse because of the abnormal shape of their genital parts like labia and irregular perineal scars or recurrent infection and pain after childbirth.

The findings indicated that genital cosmetic surgery was a solution adopted by the participants to resolve their sexual problems and sexually satisfy their husbands. Similar to these findings, Simonis et al. (2016) mentioned that one of the important reasons for women to do genital cosmetic surgery is low self-steam and self-confidence because of their genital appearance [5]. It has been evidenced that most cases of cosmetic surgeries are performed for females because they experience psychological anxiety due to rejection from their partners [15].

Also, Brotto et al. (2019) concluded psychological characteristics of women seeking FGCS (female genital cosmetic surgery) revealed that the influence of personal negative judgements and evaluations, perceived partner-related dissatisfaction, and perceived negative evaluations by them play a highly significant role in initiation of FGCS [16]. Moreover, the report of the American Society of Plastic Surgeons (2016) revealed that the number of people seeking cosmetic surgery continues to rise because they want to change their abnormality into normality [17]. Similarly, the participants in the present study complained of abnormal labia or an unattractive genital area, and cosmetic surgery helped them feel better about their genitalia.

Studies showed that most common reasons for genital cosmetic surgery is sexual dysfunction, prolapse of vagina and pain during intercourse that like present study most of their participants had such complains after vaginal delivery [17, 18]. As evidenced by the findings of the present study, congenital deformity, circumcision, and childbirth contributed to the participants' anxiety regarding the appearance of their genital areas.

In some resources, like American Society for Aesthetic Plastic surgery (2019), understanding women with sexual needs and dysfunction is an important factor before counselling for surgery because the rate of labiaplasty in the US increased more than 50% between 2014 and 2018 [18]. In comparison with the past, increasing Kurdish women’s interest in and tendency toward plastic surgery especially genital cosmetic, is an important reason to get the ideal form of their body and change their body image in order to be accepted by their partners and the society.

One motivation for performing such surgeries is to save married life, according to participants, which may cause them great anxiety if it is jeopardized. In a comparative study that was carried out by Sharp (2016), it was reported that the women in the labiaplasty group had a lower quality of married life in terms of their body image as well as an overall lower level of sexual satisfaction [19]. Research has shown that there has been an increase in genital cosmetic surgeries due to a sharp rise in the anxiety associated with perceived vulvar anomalies. Other reasons for such surgeries in the Western culture include hairless, undefined vulvas that have no protruding labia minora and irregular shape of labia minora or large labia majora [20, 21].

The results of the present study showed that some of the participants were not satisfied with the shape and size of their labia because the specific culture of the region which forced them to undergo circumcision during their childhood. But in some developed or western countries the reasons for doing such surgeries are different; some women become Barbie dolls on their own free will and preference, and this case is regarded as ‘normality’ and ‘sexy’ by their partners and the society. In this regard, women choose to undergo digital and surgical modifications to be rated as more ‘normal’ and ‘representative of society’s ideal’ [20].

This study also discovered that the majority of women with FGCS suffered perineal injuries during childbirth. Experience of vaginal tear and laceration as well as incomplete suturing the perineal wound by midwives lead to many physical and emotional complications for vulvoplasty-seeking women. On the other hand, such women become anxious not only due to physical problems like pain but also because of their inability to have normal sexual function. As reported by Brotto et al. (2019), women might seek FGCS to improve their sexual function. Moreover, sexual difficulties have been reported to affect up to a third of women across ages, and psychological factors, such as depression, anxiety and body image, are strong predictors of women’s sexual response and satisfaction especially in their married life [16].

Improving self-confidence after genital cosmetic surgery was another concept extracted from the participants’ quotations and stories. This was consistent with the study's findings, which showed that women who were engaged in a romantic relationship attempted to resolve conflicts with their partner and boost their self-confidence by undergoing labiaplasty [19]. In line with these findings, research has shown that sexual satisfaction is an important component of sexual health, sexual right, and an outcome of sexual wellbeing [22].

According to the results of a study conducted by Rowen et al. (2018), an appropriate (satisfying and safe) sexual relationship is one of the most important dimensions that has the highest effect on marital satisfaction. Thus, the improvement of sexual satisfaction could improve marital satisfaction [23]. Similarly, the results of another study conducted by Eftekhar et al. (2021) showed that gynecologic cosmetic surgery led to significant improvement in the women’s body image, sexual functioning, and sexual satisfaction. They also concluded that sexual functioning was related to and a significant predictor of both own and partner’s sexual satisfaction [24]. Moreover, Goodman et al. (2016) showed that female genital cosmetic surgery results in both ‘partner’s satisfaction’ and ‘enhancement of sexual function’. They also found positive outcomes where 97% of the women reported overall satisfaction, and a majority of them indicated sexual and emotional enhancement after female genital cosmetic surgery [25].

There are some limitations in relation to this study that are acknowledged. This study's sample size was small due to a lack of women who fulfilled our inclusion criteria and a high refusal rate among women who were eligible to participate due to privacy concerns. Another limitation was the. Finally, some of the participants were not willing to answer some of the researchers’ questions, and sometimes they provided short answers. But as a new subject, this sociocultural topic is very interesting, and further investigation into it will be helpful to improve body of knowledge in the field of women health.

## Conclusion

This study revealed that female genital cosmetic surgery improves the women’s body image and sexual function and the couples’ sexual satisfaction, especially that of husbands. Improving these factors might lead to a healthier and more pleasurable marital relationship. Quantitative studies are required to investigate the relationship between social and cultural factors and prevalence of this surgery among Kurdish. Given the male-dominated culture from which these women come, it is necessary to investigate whether they are supported with informed and independent decision making prior to undergoing surgery.

## Data Availability

All data generated or analysed during this study are included in this published article.
